# Malignant mesothelioma tumours: molecular pathogenesis, diagnosis, and therapies accompanying clinical studies

**DOI:** 10.3389/fonc.2023.1204722

**Published:** 2023-07-04

**Authors:** Ram Kumar Sahu, Sakina Ruhi, Ashok Kumar Jeppu, Husni Ahmed Al-Goshae, Ayesha Syed, Sanjay Nagdev, Retno Widyowati, Wiwied Ekasari, Jiyauddin Khan, Bedanta Bhattacharjee, Manoj Goyal, Sankha Bhattacharya, Rajendra K. Jangde

**Affiliations:** ^1^ Department of Pharmaceutical Sciences, Hemvati Nandan Bahuguna Garhwal University (A Central University), Chauras, Tehri Garhwal, Uttarakhand, India; ^2^ Department of Biochemistry, International Medical School (IMS), Management and Science University, Shah Alam, Selangor, Malaysia; ^3^ Department of Anantomy, International Medical School (IMS), Management and Science University, Shah Alam, Selangor, Malaysia; ^4^ Department of Anatomy, Physiology, and Biochemistry, Management and Science University, Shah Alam, Selangor, Malaysia; ^5^ Department of Pharmacy, Gyan Ganga Institute of Technology and Sciences, Jabalpur, Madhya Pradesh, India; ^6^ Department of Pharmaceutical Sciences, Faculty of Pharmacy, Universitas Airlangga, Surabaya, Indonesia; ^7^ School of Pharmacy, Management and Science University, Shah Alam, Selangor, Malaysia; ^8^ Girijananda Chowdhury Institute of Pharmaceutical Science, Tezpur, Assam, India; ^9^ School of Pharmacy & Technology Management, SVKM’s NMIMS, Shirpur, MH, India; ^10^ University Institute of Pharmacy, Pt. Ravishankar Shukla University, Raipur, Chhattisgarh, India

**Keywords:** mesothelioma, asbestos, SV40, biomarkers, hippo signaling pathway

## Abstract

The pathetic malignant mesothelioma (MM) is a extremely uncommon and confrontational tumor that evolves in the mesothelium layer of the pleural cavities (inner lining- visceral pleura and outer lining- parietal pleura), peritoneum, pericardium, and tunica vaginalis and is highly resistant to standard treatments. In mesothelioma, the predominant pattern of lesions is a loss of genes that limit tumour growth. Despite the worldwide ban on the manufacture and supply of asbestos, the prevalence of mesothelioma continues to increase. Mesothelioma presents and behaves in a variety of ways, making diagnosis challenging. Most treatments available today for MM are ineffective, and the median life expectancy is between 10 and 12 months. However, in recent years, considerable progress has already been made in understanding the genetics and molecular pathophysiology of mesothelioma by addressing hippo signaling pathway. The development and progression of MM are related to many important genetic alterations. This is related to NF2 and/or LATS2 mutations that activate the transcriptional coactivator YAP. The X-rays, CT scans, MRIs, and PET scans are used to diagnose the MM. The MM are treated with surgery, chemotherapy, first-line combination chemotherapy, second-line treatment, radiation therapy, adoptive T-cell treatment, targeted therapy, and cancer vaccines. Recent clinical trials investigating the function of surgery have led to the development of innovative approaches to the treatment of associated pleural effusions as well as the introduction of targeted medications. An interdisciplinary collaborative approach is needed for the effective care of persons who have mesothelioma because of the rising intricacy of mesothelioma treatment. This article highlights the key findings in the molecular pathogenesis of mesothelioma, diagnosis with special emphasis on the management of mesothelioma.

## 1 Introduction

It has been shown that the risk of developing malignant mesothelioma (MM) is significantly increased if a person has been exposed to asbestos. Patients with MM have an extremely poor diagnosis when the disease is discovered, and their life expectancy is only seven to twelve months from the time the disease is detected (1). The latency period for MM tumour development after asbestos exposure is often 30-40 years, suggesting that many genetic and epigenetic changes must accumulate before the disease manifests. On the other hand, nothing is known about the molecular pathogenesis of MM (2). Malignant pleural mesothelioma (MPM), as the name implies, is a malignancy of the mesothelium layer of the pleural cavities of the lungs. Pleural mesothelioma is a fatal and incurable cancer that originates in the cells lining the serosal membrane of the pleural cavity (3). This disease has a dismal prognosis. The average maturity of this disease at diagnosis is 75 years old. The number of deaths from MPM continues to increase despite the increasing ban on the manufacture and supply of asbestos in recent decades (4). Among the three forms of mesothelioma, epithelioid (60 percent) and sarcomatoid (20 percent) are the most common. Almost all cases of mesothelioma may be traced back to asbestos exposure, a relationship that was discovered by South African pathologist, Wagner, back in 1960 (5). Pleural mesothelioma was becoming more common in parts of the Cape asbestos field where Cape Blue asbestos was mined (crocidolite asbestos) (6). Mesothelioma is directly causally related to occupational asbestos exposure, which provides some accuracy in predicting future occurrence (7).

MPM patients have limited treatment options because the disease is usually discovered at a late stage. Apart from surgery, MPM can be treated with chemotherapy, radiation therapy, or a combination of these techniques. The clinical stage and characteristics of the patient determine the appropriate therapy (8). Extra pleural pneumonectomy (EPP), which removes the diseased pleura and lung as well as the pericardium and half of the diaphragm, or radical pleurectomy/decortication (P/D) are the two surgical options for patients eligible for macroscopic surgery (9). As surgical microscopic clearance is difficult, surgical treatment is usually coupled with chemotherapy and/or radiation. Although many patients are not always surgical candidates, systemic chemotherapy is the most common treatment option. Cisplatin and pemetrexed are currently the only FDA-approved MPM chemotherapy medicines. They have been used as standard therapy for MPM for more than a decade (10). There are no approved second-line therapies for MPM. The combination of cytostatics with anti-VEGFA therapy has shown potential. Bevacizumab (MAPS study) and nintedanib (LUME-Meso study) have shown promising results in clinical trials when added to chemotherapy (11). The PD-L1 and PD-L2 are naturally occurring ligands that are repeatedly expressed over tumor cells as well as the microenvironment surrounding them (12). Tumor inhibition can be enhanced by inhibition of PD-L1 ligand interaction with PD1 receptor of T-cell. It is possible to target these receptors with the application of the monoclonal antibodies that inhibit the PD-1/PD-L1 pathways (13).

The tumor suppressor gene BRCA1-associated protein 1 (BAP1), which is encoded on chromosome 3p21.1, produces a deubiquitinases enzyme that controls a range of physiological processes, including apoptosis, DNA repair response, cellular advancement, growth inhibition, and chromatin remodeling (14). Decreased concentrations of the functional protein driven on by germline BAP1 alterations promote retention of genomic alterations and finally cancer. Recently, BAP1 depletion has proved a nearly 100% predictive indicator of cancer in mesothelial differentiation (15).

Fluorescence in situ hybridization (FISH) was executed to reveal a homozygous mutation of CDKN2A (encoded on p16 protein) is the forthcoming effective screening investigation in mesothelial differentiation with no apparent morphological or BAP1 deletion. Similarly, to BAP1 deletion, CDKN2A homozygous mutation is practically 100% tailored for malignant mesothelioma but just 48-88% susceptible to the detection of pleural mesothelioma, with the majority of values in the spectrum of 50-65%, as well as increasing to 80-100% responsive for the evaluation of sarcomatoid mesothelioma. The detection limit of CDKN2A FISH reduces in the peritoneum by approximately 25-29% (16).

Approximately half of all MM tumours contain mutations in the gene encoding the tumour suppressor protein neurofibromatosis type 2 (NF2). Merlin is an Ezrin/radixin/moesine family protein encoded by NF2. From Drosophila to mammals, Merlin acts as an upstream regulator of the Hippo signaling cascade (17). Signaling mediated by hepatomegaly (Hippo) is a critical controller of organ size, organogenesis, and stem cell self-renewal (18). As a result, the Hippo signaling system was newly involved in the process of tumour growth. Various biomarkers, such as soluble mesothelin, osteopontin, fibulin-3, HMGB1, ctDNA, heparanase, and STAT3 have been intensively studied for their diagnostic potential in MPM. It seems to be a possible research direction to search for biomarkers useful for the early diagnosis and treatment of MM (19).

## 2 Pathogenesis

### 2.1 MM progression by asbestos

Mesothelioma was originally described in 1931, but it wasn not until 1960 that South African epidemiological research linked it to asbestos exposure (20). It is now known that mesothelioma in men is caused by occupational asbestos exposure in 85 percent of cases, while only 10 percent of individuals with asbestos exposure develop mesothelioma (21). The possibility of acquiring lung cancer increases tenfold to one hundredfold when asbestos exposure is combined with cigarette smoking. This is true compared to people who have not been exposed to asbestos. In contrast, there is no evidence that smoking enhances the likelihood of getting mesothelioma (22).

Asbestos fibers have been shown to affect mitotic activity. Mesothelioma may be caused by fibers that tear or puncture the mitotic spindle, interrupting mitosis. As a third explanation, harmful oxygen radicals are produced. Reactive oxygen species (ROS) that are iron-related cause DNA damage and DNA strand break in cells when asbestos fibers are inhaled (23). A persistent kinase-mediated signaling mechanism is the fourth. In this connection, asbestos fibers were observed to trigger mitogen-activated protein kinases (MAPKs) and extracellular signal-regulated kinases 1 and 2 in mesothelial cells, leading to early response protooncogenes (24). In addition, platelet-derived growth factor β and transforming growth factor have been found to be the only growth factors that have been shown to prevent the mesothelioma growth. Despite the reality that various growth factors and even their receptors play a role in the dissemination of mesothelioma (25).

Mesothelioma has a complex etiology. Human mesothelial cells exposed to crocidolite asbestos fibers *in-vitro* become hazardous in a dose-dependent way (26). There is evidence that crocidolite can cause macrophage build-up in the pleura and the lung Tumour necrosis factor (TNF)-α is released by macrophages in response to crocidolite exposure, and TNF-α receptors are present in human mesothelial cells. Further, the human mesothelial cells also produce TNF-α as a result of asbestos exposure (27). To achieve resistance to apoptosis, TNF-α receptor and ligand contact stimulates NF-κB signaling, allowing human mesothelial cells to divide instead of dying (28). Asbestos can induce mesothelioma if sufficient DNA abnormalities are present.

The various connection between asbestos exposure and mesothelioma are explained by several alternative pathogenetic pathways. There are two main pathways by which asbestos fibers can cause mesothelioma: by inhalation of more than 5 μm thin asbestos fibers that penetrate the lung epithelium and enter the pleural space of the lung (29). First of all, the generation of oxidative radicals leading to DNA damage and mutations is associated with phagocytosis of asbestos fibers, which in turn produces oxygen-free radicals (30). Asbestos fibers have been shown to phosphorylate and generate many pro-oncogenic protein kinases in mesothelial cells, in addition to interfering with mitosis (31). Malignant cells can utilize mesothelial cells to secrete platelet-derived growth factor (PDGF), vascular endothelial growth factor (VEGF), and inflammatory tumor growth factor (ITGF) (32). The pathogenesis of mesothelioma is likely to include factors that are unique to the host as well as those that are universal.

Mesothelioma is thought to be caused by some mechanisms triggered by exposure to asbestos (33–36), which include the following:

➢When fibers are inhaled, cycles of tissue damage and repair and local inflammation are repeated.
➢Mesothelial cells are directly damaged by asbestos fiber inhalation, resulting in chromosomal abnormalities.
➢When asbestos-exposed mesothelial cells release inflammatory cytokines and growth factors, a tumor-friendly microenvironment is created.
➢Macrophages not only phagocytose asbestos fibers, but also produce reactive oxygen species (ROS) that can cause intracellular DNA damage and defective repair.
➢High Mobility Group Box 1 is released by asbestos-induced death of mesothelial cells, further promoting and maintaining chronic inflammation
➢Increasing the expression of proto-oncogenes by phosphorylating protein kinases promotes abnormal cell growth.

### 2.2 MM progression influenced by SV40

In the 1950s and 1960s, polio vaccines contaminated with Simian virus 40 (SV40), a DNA cancer virus, were thought to have infected people (37). Even then, SV40 was found in people who had not received the vaccine, suggesting that there are other ways to become infected. SV40 has been associated with malignant mesothelioma. There are several types of polyomaviruses, including SV40, which is an oncogene in human and rat cells because it suppresses the expression of tumor suppressor genes (38). Cancers of the brain and bones, lymphoma, and malignant mesothelium are all known to be caused by this virus, as are atypical proliferations and superficial noninvasive lesions of the mesothelium that contain the SV40 DNA sequence (39). Human cell lines can be infected with this virus, and its DNA can be found in up to 60% of human mesotheliomas. Scientists have demonstrated that malignant cells and reactive mesothelial cells contain SV40, but not normal tissue or lung cancer. This virus was widely distributed in the 1950s and 1960s as part of the Salk polio vaccine (37). It can also be transmitted horizontally to humans. However, there is no conclusive evidence of an association between vaccine-triggered infection and mesothelioma cases. In general, the influence of SV40 on the development of mesothelioma has not yet been established. Nevertheless, asbestos is still the main cause of this disease (39).

## 3 Understanding mesothelioma’s molecular pathogenesis

### 3.1 Addressing hippo signalling pathway

The Hippo signalling pathway of Drosophila is essentially identical to that of mammals. This similarity was first noted in the genetic search for overshooting mutations in Drosophila. These essential cascades controlling autophagy, immunity, energy stress, and DNA damage contain upstream regulators (MST1/2 and LATS1/2), scaffold/adaptor proteins (SAV1 and MOB1), and two final transcriptional coactivators (YAP/TAZ) (40). Major effectors YAP and TAZ are either retained or degraded in the cytoplasm as a result of LATS1/2 phosphorylation, or sometimes they get dephosphorylated and translocated to the nucleus, therefore activating their transcriptional activity (41). Molecular research in animals and humans has shown that there is an inherited predisposition to asbestos-related cancer. According to conventional cytogenetic studies, most mesotheliomas have an aberrant karyotype, often with significant aneuploidy and structural rearrangements. However, rearrangements of 1p, 3p, 9p, and 6q are also frequent. It has been observed that all mesothelioma cell lines have lost their heterozygosity due to neurofibromatosis type 2 (NF2). The observation that deletion of P16INK4A, P14ARF, and NF2 are the most common irregularities suggests that mesothelioma growth requires a specific sequence of tumour suppressor gene deletion. It may be possible to determine the sequence of these events in mesothelioma formation using new animal models (42).

### 3.2 Inhibition of the hippo pathway in mesothelioma fibroblasts

**Figure 1:**
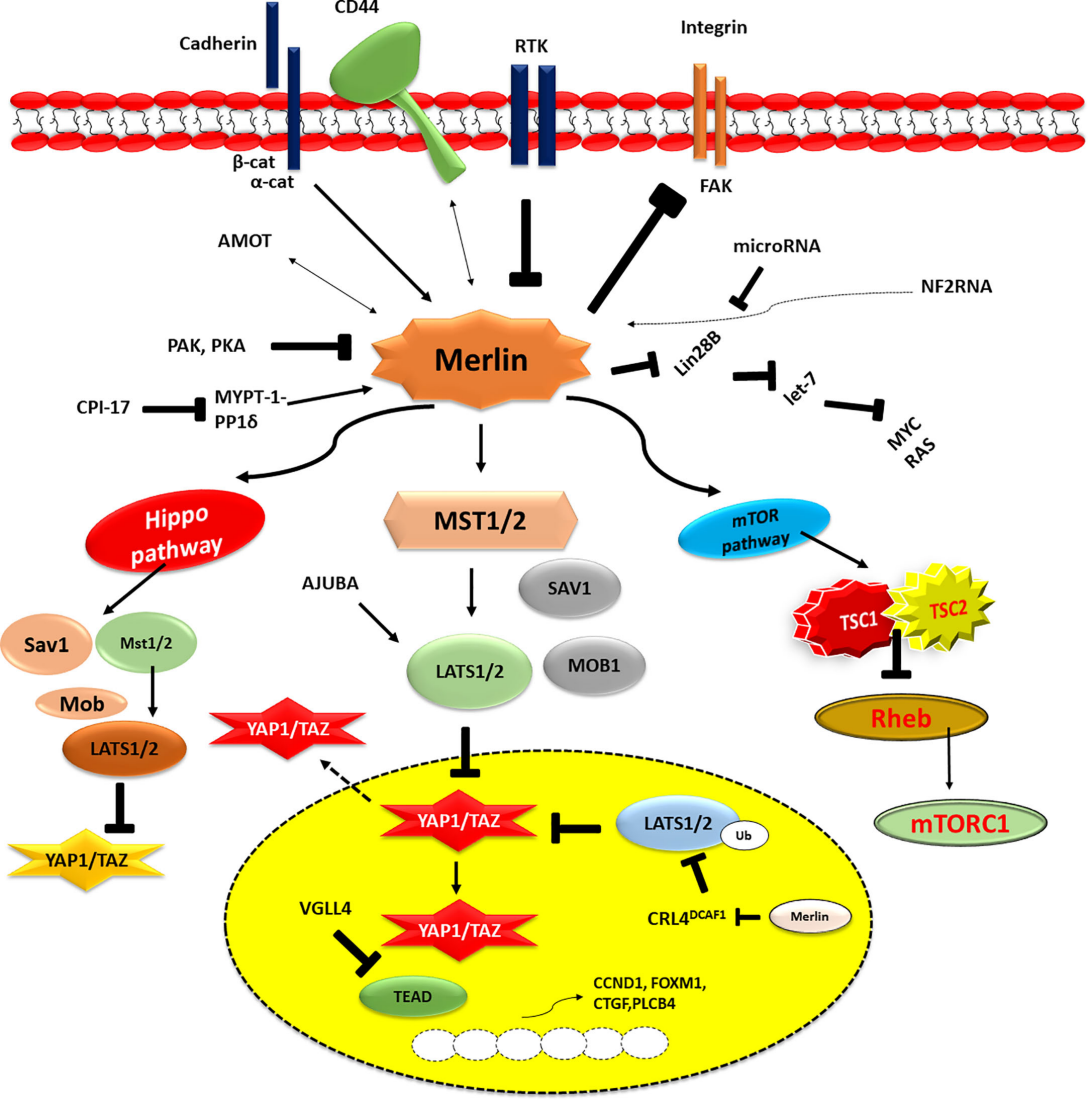
The above figure illustrates the dysfunction of the NF2/merlin-Hippo signalling cascades in malignant mesothelioma cells. This activity is influenced by signals from the extracellular environment, which are sent by cell-to-cell contact (cadherin), cell-to-matrix contact (CD44 and integrin), and/or growth factor receptors (RTKs). Underphosphorylated merlin controls Hippo signalling cascade and YAP1/TAZ transcriptional coactivator activity when it is activated. YAP1/TAZ transcriptional coactivators are underphosphorylated (activated) in MM cells due to the frequent alteration of merlin (the NF2 gene product) and Hippo pathway components, including LATS1/2. As a result, many pro-oncogenic genes, including CCDN1, FOXM1, CTGF, and PLCB4, are expressed in MM cells. P, phosphorylation; Ub, ubiquitination.

Moesin-ezrin-radixin-like protein (Merlin) has a genetic mutation that leads to neurofibromatosis type 2 (NF2). Merlin is a component of the ring 4.1 group of cytoskeletal linkage proteins (**Figure 1**). One of the most significant and critical downstream signaling cascades of Merlin is the Hippo signaling system. This is involved in critical biochemical functions, including control of organ size and growth, differentiation, tissue formation (restriction of cell growth), modulation of cell division, cell death, and tumor progression (43). The following four components, SAV1 (also known as WW45), MST1 and MST2 kinases, MOB1, and LATS1 and LATS2 kinases, have been demonstrated to function together as tumour suppressors. When MST1/2 kinases (complexing with the SAV1 scaffold protein) receive upstream signals, they phosphorylate and activate LATS1/2. MOB1 scaffold protein activates the latter, which subsequently phosphorylates and inactivates transcriptional coactivators YAP1 and TAZ. In the cytoplasm, the phosphorylated YAP1/TAZ are then either maintained (through contact with 14-3-3) or destroyed (via polyubiquitination) (44). A lack of the Hippo pathway’s activation enables the YAP1/TAZ to get enter the nucleus and serve as the coactivators for the transcription of genes. The transcription factor YAP1/TAZ interacts with TEA domain transcription factors, SMADs, T-Box 5 (TBX5), Runt related transcription factor (RUNX), p73, early growth response-1 (EGR1) and the carboxyl-terminal fragment of Erb-B2 receptor tyrosine kinase 4 (ERBB4) transcription factors (45). Through its actions on cell junction-associated proteins which impart a chief role in the regulation of the Hippo signaling, Merlin stimulates the Hippo pathway and inhibits YAP1/TAZ as well (46). Among other intracellular CD44 domain proteins and ERM proteins, the protein known as Merlin suppresses cell development by binding to mature adherens and tight junctions, where it specifically targets angiomotin and beta-catenin. MST1/2 and LATS1/2 are thought to act as scaffolds for MST1/2 and LATS1/2 as well as bind to and suppress the activity of YAP1. On the other hand, the nature of the link that exists between angiomotin and merlin in the Hippo pathway is still a mystery (47). In addition, angiomotin is assumed to provide an activator of Merlin, allowing it to better connect with its target, LATS1/2.

Sekido et al. in 2018 described the efficiency of the pro-oncogenic activities of TAZ in MM. In a study using MM cell lines, TAZ activation was shown to be higher in MM cells compared to immortalized mesothelial cells. Suppression of TAZ in MM cells by shRNA was shown to significantly slow proliferation, cell motility, invasiveness, and anchorage-independent growth. Microarray studies were used to determine the reduction in activation of the target genes of TAZ in mesothelioma cells. It was found that YAP- and TAZ-activated cells elevated most genes, although TAZ upregulated cytokine genes and their receptors more than YAP. When TAZ and TEAD transcription factors bind to the promoter region of the IL1β gene, they increase its transcription rate and induce the cell proliferation. In contrast, the IL -1 receptor antagonist or IL1β knockdown decreased cell growth, suggesting that IL -1 signal suppression may have more repressive effects on MM cells actively expressing via TAZ than previously thought. It is proposed that MM cells develop and retain their malignant properties via a TAZ-IL-1-β-axis combination (48).

## 4 Epidemiology

Because of the widespread application of asbestos since World War II, the number of cases and deaths from mesothelioma began to increase in the 1960s (49). Mineral fibers, known as asbestos, are used in a variety of commercial applications and are the primary cause of mesothelioma (50). These six mineral fibers are crocidolite, actinolite, tremolite, anthophyllite, amosite, and chrysotile. It is possible to freely use the other 400 different fibers because they are not regulated. There are two primary adverse effects of asbestos: fiber size and biopermanence (51). Regulated and unregulated fibers are found in almost all geologic formations, and human activities (new site development, mining, and vehicular activities) emit fibers into the air and expose humans to the environment (52).

**Figure 2:**
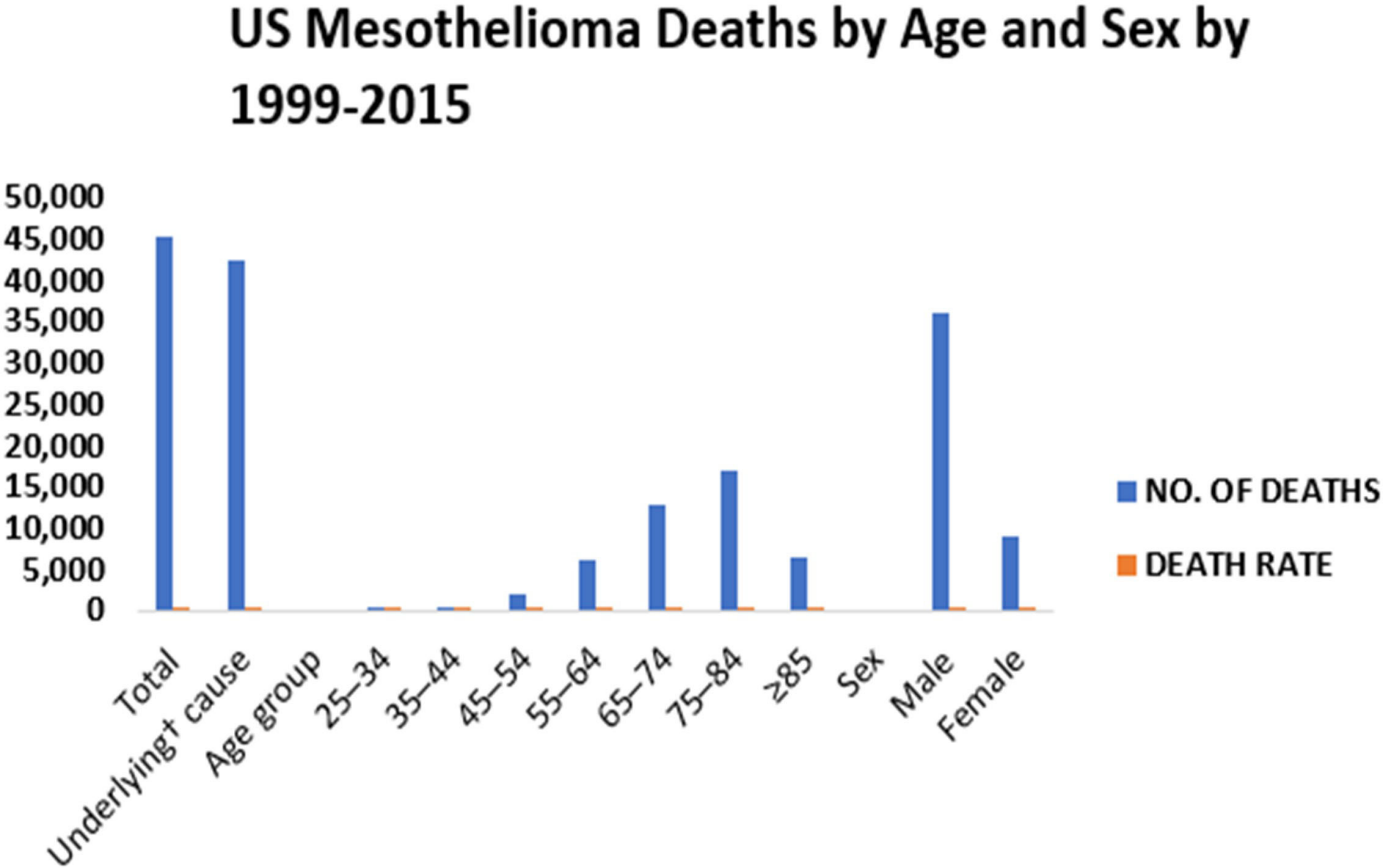
Epidemiology of mesothelioma.

In certain cases, non-regulated asbestos fibers are more hazardous than six regulated asbestos fibers. According to Metintas et al, 2022, there were a total of 45,221 deaths due to malignant mesothelioma between the years 1999 and 2015 (53). The number of deaths caused by mesothelioma has increased in people younger than 85 years, men and women, people of black, white, Pacific Islander or Asian descent (54). The recurrence of malignant mesothelioma mortality in people under the age of 55 implies that asbestos fibers and perhaps other EMPs are still being inhaled. In the United States, mesothelioma affects about 3,000 people each year(55). The mortality rate for men was 24.9 deaths per million between 1999 and 2015 (**Figure 2**), while the mortality rate for women was 4.65 deaths per million between 1999 and 2015 (https://www.asbestos.com/mesothelioma/death-rate/).

According to the World Health Organization (WHO), the United States had the greatest age-standardized incidence rates in 2018 (56). In addition, an increase in MM patients due to asbestos exposure has been observed in other developed countries such as Australia, New Zealand, and the United Kingdom (8). In the same age group in Europe, the rate is 0.6 per 100,000 for men and 0.4 per 100,000 for women, with incidence rates (IRs) of 1.4 per 100,000 for men and 0.6 per 100,000 for women from age 50-54 (Alpert, van Gerwen and Taioli, 2020). IRs in Europe in 3.4/100,000. The mean age at diagnosis was 61.1 years, and 63.2% of patients were male. 78.5% had epithelioid malignancies, 38.7% had asbestos exposure prior to diagnosis, and 62.3% had stage III and 37.7% had stage IV malignancies (57).

## 5 Symptoms

Mesothelioma is sometimes mistaken as a more prevalent disease. Cough, shortness of breath, chest or abdominal discomfort, and fluid build-up are some of the symptoms. Chest discomfort and shortness of breath are among the symptoms of pleural mesothelioma. Bloating and stomach discomfort are two of the symptoms of peritoneal mesothelioma (58). Asbestos exposure also takes years to develop, making it difficult to identify it early. Approximately 70% of patients report shortness of breath and chest discomfort or shortness of breath owing to pleural effusion in the early stages (59). Medical intervention or obliteration of the pleural cavity by a tumor tends to reduce the amount of fluid in the pleura. In addition to dyspnea, discomfort often occurs as the disease growth across the pleural surface due to the limited ability to breathe and the encasement by the thoracic tumor (60).

Although less prevalent, pleural chest discomfort irritation tends to develop with illness progression, particularly in the case of an invasion of the chest wall. These include secondary bone pain with chest invasion and neuropathic pain and damage to the intercostal nerves (61). Other symptoms of malignant pleural mesothelioma (MPM) include fatigue, loss of appetite, weight loss, sweating, and pain. Cytokines are produced by the tumor and the host in response to cytokines in the blood (62). There is a lower incidence of bronchial tumor-related symptoms, such as cough, hemoptysis, and lymphadenopathy, in MPM. Invasion of the superior vena cava, paralysis, or laryngeal nerve dysphasia may result from the local cancer invasion (63). In many cases, the latter is a pre-event -terminal. In other cases, the patient is asymptomatic, but an anomaly was identified in an imaging study conducted for another cause (64). As a result of early diagnosis, asymptomatic individuals tend to have a higher life expectancy. For this reason, individuals with pleural effusion and a history of asbestos exposure should be closely monitored, even if the effusion is minor or resolves spontaneously. As a result of active monitoring, a greater percentage of these individuals will be diagnosed with MPM sooner (65).

## 6 Diagnosis

A person working in the asbestos industry is a good indicator of exposure to the harmful chemical. Accurate diagnosis of mesothelioma depends on obtaining sufficient tissue for pathology (66). In patients with pleural effusions, a sample of the fluid should be obtained and examined cytologically as the first step in making a diagnosis. In most cases, the cells in the fluid are malignant, and the diagnosis of mesothelioma can be accepted if the clinical, radiologic, and cytologic data are consistent (67). However, cytology of pleural fluid and pleural biopsy alone to identify the tissue are rarely suggested for a definitive diagnosis. When cytologic examination of pleural fluid is inadequate, computed tomography with enhanced contrast (CT) is required to both determine the extent of disease and guide percutaneous biopsy. Patients should undergo radiologic imaging, as it provides valuable information regarding diagnosis and stage of development.

Individuals with MPM generally have high levels of the blood protein known as the cancer antigen (CA)-125, which is utilized as a diagnostic biomarker. Approximately 1/3 of women with MPM are initially misdiagnosed with ovarian cancer, as ovarian cancer is more common and is known to correlate with elevated CA -125 (68). Modern Calretinin antibodies need both cytoplasmic and nuclear labeling in order to establish mesothelioma detection. Calretinin has a detection accuracy of 81-100% in edema for mesothelioma (69).

Different biopsy techniques are used depending on the location and type of disease, the patient’s ability to undergo surgery or invasive treatment, and the availability of medical resources (70).

## 7 Radiological results

Mesothelioma must be diagnosed, staged, and treated using radiological imaging. X-rays, CT scans, MRIs, and PET scans have all been used to diagnose the disease.

### 7.1 Computed tomography

Contrast-enhanced intravenous I n the case of suspected pleural malignancy, CT is the main imaging modality. C-T scans allow for visualizing a patient’s whole pleural surface and diaphragm, as well as the mediastinal nodes (71). Besides liver and adrenal glands, the scan should also encompass the lower abdomen and pelvis if there is a history of abdominal or pelvic cancer. Pneumoconiosis is a difficult condition to treat. The CT features most helpful in diagnosing malignant pleural disease are pleural thickening of > 1 cm, nodular pleural thickening, and mediastinal pleural involvement. The specificity of each of these observations ranged from 1100%, 94%, and 88%, respectively. Sensitivity was ranged from 41, 51, 36, and 56%, respectively (72). Patients with malignant mesothelioma and bilateral pleural calcification on CT are rare. In contrast, the results of other studies using CT scans showed a considerable decrease in thoracic volume. They have a high capacity for accurate prognosis, but their absence does not exclude the possibility of pleural carcinoma (73).

### 7.2 Magnetic resonance imaging

Screening for malignant mesothelioma with MRI is not routinely performed; however, MRI can offer extra staging information over and beyond CT in individuals with potentially significant illnesses (Lopci, Castello and Mansi, 2022). This technique allows doctors to better identify tumors that have spread into the diaphragm or chest wall using gadolinium enhancement and magnetic resonance imaging (MRI). For people who are not allowed to receive intravenous iodine-containing contrast, MRI is the tool of choice (74).

### 7.3 Positron emission tomography

At PET, the normalised uptake value (NUV) is a semiquantitative assessment of the metabolic activity of a lesion. When compared to the NUV of various benign pleural illnesses, including inflammatory pleuritis or pleural plaques, the NUV of malignant pleural mesothelioma was significantly higher. Because of its ability to provide information about metabolism and anatomy, a PET scan can be used to grade mesothelioma before surgery (75). On the other hand, there is validation that fluctuations in fluorodeoxyglucose (FDG) absorption within the tumor may signal a response to treatment. This suggests that PET could be used to assess the efficacy of chemotherapy, chemoradiation therapy, and possibly even other treatments (76).

### 7.4 Imaging and staging

The International Mesothelioma Interest Group (IMIG) TNM classification system is used for evaluating individuals with possibly resectable illness and is not fully relevant to imaging (77). Malignant mesothelioma may be staged using CT and MRI, which have equal overall accuracy. However, both methods may underestimate stage disease. When it comes to cancer resectability, both methods have been proven to be accurate, although CT is more often employed due to its speed and availability (78). Entrance into therapeutic trials may be contingent on radiological staging. As with lung cancer staging, mediastinal lymph nodes are frequently implicated in mesothelioma; CT has poor accuracy in detecting an invasion of mediastinal lymph nodes (79). Although a high NUV correlates with the presence of N2 illness, FDG PET appears to be rather ineffective in distinguishing between mediastinal lymph node metastases and mediastinal pleural involvement. It may be reasonable to combine scans from CT and PET in the evaluation of these patients, although studies are limited. Normally, a mediastinoscopy is performed before major surgery to rule out N2 illness. N3 or metastatic illness that is occult may be detected with CT-PET (80).

## 8 Multiple therapies for mesothelioma management

**Figure 3:**
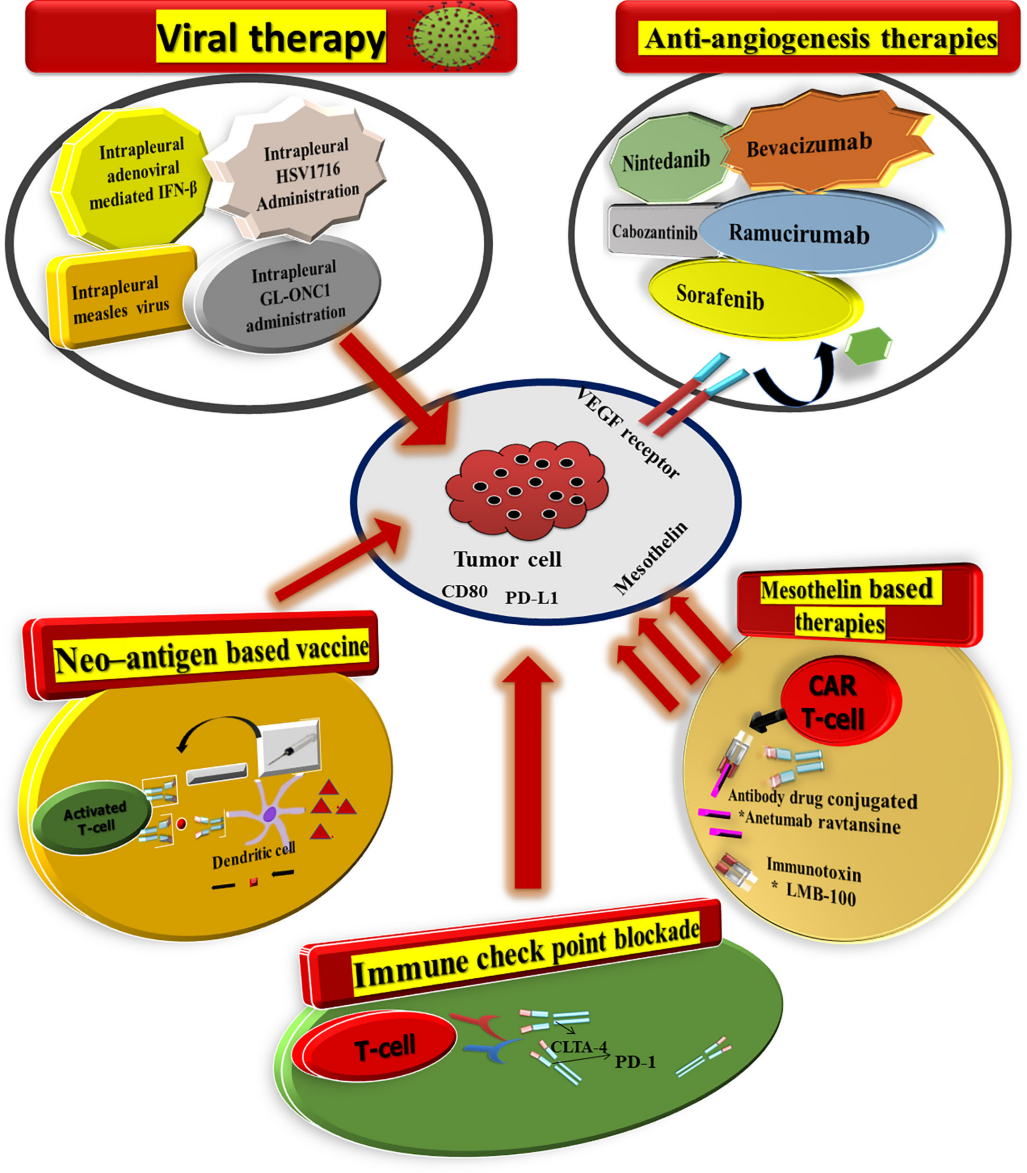
There are a plethora of therapeutic approaches for mesothelioma now being investigated in the clinic. Furthermore, Nintedanib also targets the fibroblast growth factor receptor and platelet-derived growth factor receptor, in addition to VEGF.

Mesothelioma can be treated with a mixture of an individual (chemotherapy) or combination therapy (radiotherapy, targeted therapy) (multimodality treatment). Early diagnosis and treatment are key factors in the success of mesothelioma surgery (**Figure 3**).

### 8.1 Palliative care of mesothelioma

A competent palliative care program may assist manage physical symptoms as well as provide psychological, emotional, or spiritual support, according to the European Respiratory Society, British Thoracic Society, and International Mesothelioma Interest Group (81). A palliative (supportive) treatment differs from hospice care. Even when combined with other types of therapy such as chemotherapy, radiation, and immunotherapy, palliative care enhances the quality of life and comfort of mesothelioma patients, according to a recent multicentre randomized controlled research (82). Palliative treatment is given to patients who are malnourished and in poor health, have mesothelioma in Stage III or IV, or who have biphasic or sarcomatoid mesothelioma (83).

### 8.2 Surgery

Surgical intervention is controversial and should be performed only in individuals in the early stages of the disease and with good functional status. Surgical therapy for mesothelioma began in the mid-20th century with a procedure used to treat tuberculous empyema patients (84). Pneumonectomy decortication (P/D) or extrapleural pneumonectomy (EPP) were other names for the procedure. In the 1970s, this procedure was performed to treat mesothelioma, cancer caused by asbestos exposure. Cancer stage, performance status, histology, and nutrition are all factors in determining a patient’s surgical candidacy for thoracic surgery (85). If the patient is Stage I or II palliative care, surgery can be used. There are very few cases in which mesothelioma is treated surgically since the disease is diagnosed at Stage III or IV. Palliative treatment is the use of surgery to reduce the tumor mass and relieve symptoms (86).

### 8.3 Chemotherapy

To limit the development of cancer cells, by either killing them or preventing them from dividing, cancer patients may receive chemotherapy by ingestion, injection, or infusion or by topical application using drugs or chemicals. Treatment, such as surgery, radiation therapy, or biological therapy may be combined with it (87).

### 8.4 First-line chemotherapy

Anthracyclines and taxanes have been used as single agents in studies of chemotherapy. Alkylating drugs and topoisomerase inhibitors were also used. In the first randomized trial, treatment-naïve subjects were randomly assigned to receive either pemetrexed or cisplatin (88). According to the results of this research, the pemetrexed group had a median survival of 12.1 months, but the cisplatin group had a median survival of only 9.3 months. After the additional administration of folic acid and vitamin B12, the rate of toxicity decreased significantly. The results of this experiment led to pemetrexed being granted approval by regulatory authorities around the world for use in conjunction with cisplatin in the treatment of MPM. Since then, pemetrexed has been the standard first-line chemotherapy for patients diagnosed with MPM. Based on phase II trials that indicated better response rates with gemcitabine, vinorelbine, or anthracyclines, single-agent chemotherapy is now widely accepted (89).

### 8.5 First-line combination chemotherapy

For Combination, chemotherapy is the most effective way to treat mesothelioma because it produces greater therapeutic results than single-agent chemotherapy. The combination of cisplatin and anti-folates (such as pemetrexed) is the most frequently utilized regimen for patients with unresectable MPM in the advanced stages. The FDA approved cisplatin with pemetrexed based on the phase III EMPHACIS study published by Vogelzang et al. (90).

### 8.6 Single-agent chemotherapy for second-line treatment

After initial therapy for malignant pleural mesothelioma, most patients are candidates for further chemotherapy. Unfortunately, there is insufficient clinical evidence to make a judgment on whether or not patients can be restarted on antifolate after a prolonged break from platinum-pemetrexed-based treatment (first-line). In the second clinical trial, raltitrexed and cisplatin were studied alongside cisplatin alone. The survival benefit of raltitrexed/cisplatin was equivalent to that of pemetrexed (11.4 months versus 8.8 months), but response rates were lower (91). Because of its limited impact on clinical trials, this experiment was considered of little significance. Neither the European Medicines Agency (EMA) nor the United States Food and Drug Administration (USFDA) have given their approval for the use of raltitrexed for the management of MPM (92). Because it is usually better tolerated, carboplatin can be substituted for cisplatin in the elderly (in whom cisplatin is toxic). Second-line treatments include irinotecan-cisplatin-mitomycin, cisplatin-gemcitabine, and oxaliplatin-raltitrexed (93).

### 8.7 Radiotherapy

Radiation is utilized to treat MPM patients. Although radiation is seldom used to treat individuals with malignant pleural mesothelioma, it is used to relieve pain caused by the invasion of the chest wall. The whole hemithorax, the thoracotomy incision, and the locations of the chest drains were exposed to a 54Gy dose of radiation (94).

#### 8.7.1 Prophylactic radiotherapy

As a consequence of the outcomes of the SMART and PIT studies, the American Society of Clinical Oncology no longer recommends radiotherapy of the chest wall tracts following an operation to avoid seeding of the tumor parietal. Radiosensitivity of mesothelioma has been established *in-vitro* and in animal models through research. The utility of radiation treatment in MPM has therefore been clinically proven (95).

#### 8.7.2 Palliative radiation

Pain control, dysphagia therapy, and superior vena cava compression have all been treated with palliative radiation in MPM. In the radiation field, the local control and survival were excellent, with a recurrence rate of only 12 percent. A trimodality strategy with induction chemotherapy, radical hemi thoracic radiation, and EPP was developed as a result of these findings (96). Radical hemi thoracic radiation has been used in two new prototypes (1) the use of radical hemi thoracic radiation as part of a lung-sparing multimodality approach (IMPRINT = intensity-modulated pleural radiation treatment) and SMART (surgery for mesothelioma following radiation therapy) have both employed extreme hemi thoracic radiation in their new prototypes. There are two techniques for radiation treatment that both rely on intensity-modulated radiation therapy (97).

### 8.8 Targeted therapy

#### 8.8.1 Anti-angiogenic drugs

VEGF imparts an essential function in MPM by increasing angiogenesis and encouraging tumor development. Investigations into the use of antiangiogenic tyrosine kinase inhibitors (TKI) for the treatment of MPM are now being conducted either alone or in combination with standard therapy (cisplatin + pemetrexed) or ongoing therapy, or both. In addition, an anti-VEGF monoclonal antibody called bevacizumab has been validated for the treatment of MPM (98).

#### 8.8.2 Immunotherapy

Rather than treating cancer cells directly with drugs, immunotherapy is an extremely feasible approach for mesothelioma treatment because it induces an immunological response against the tumour by stimulating the immune system. This is in contrast to conventional cancer treatments that target the cancer cells themselves (99). Immunotherapy may be a viable option for MPM patients, given that lymphocyte infiltration inside the tumor mass has been linked to a better prognosis. Cytotoxic CD8 T cells enhanced the survival of individuals with malignant pleural mesothelioma (tumor-infiltrating lymphocytes) (100). Various treatments can be used to restore the anti-tumour immune response, focusing on the immunosuppressive microenvironment of the tumour. Patients suffering from MPM taking these drugs may experience different effects at different stages of the anti-tumour immune response.

#### 8.8.3 Virotherapy for MPM

Infection of tumor cells with viruses can induce an immune response against the tumor (viro-immunotherapy). The viruses impart therapeutic effects and alter the infected tumor cells by modulating the gene transfer (101). There have been clinical studies on oncolytic viruses, comprising vaccinia virus, reovirus, herpes simplex virus (HSV), measles virus, adeno-associated virus (ADV), and others. Recombinant replication inadequate ADV has been the most commonly utilized viral vector in MPM. Moreover, various phase I/II trials have used ADV vectors that encoded the suicide gene HSV thymidine kinase (Ad. HSVtk) in combined application with the gancyclovir (intravenous delivery) and interferon α (Ad.IFNα) or interferon β (Ad.IFNβ) solely or in mixture with chemotherapy and cyclooxygenase inhibition (102).

#### 8.8.4 Recurrent mesothelioma biomarkers

**Figure 4:**
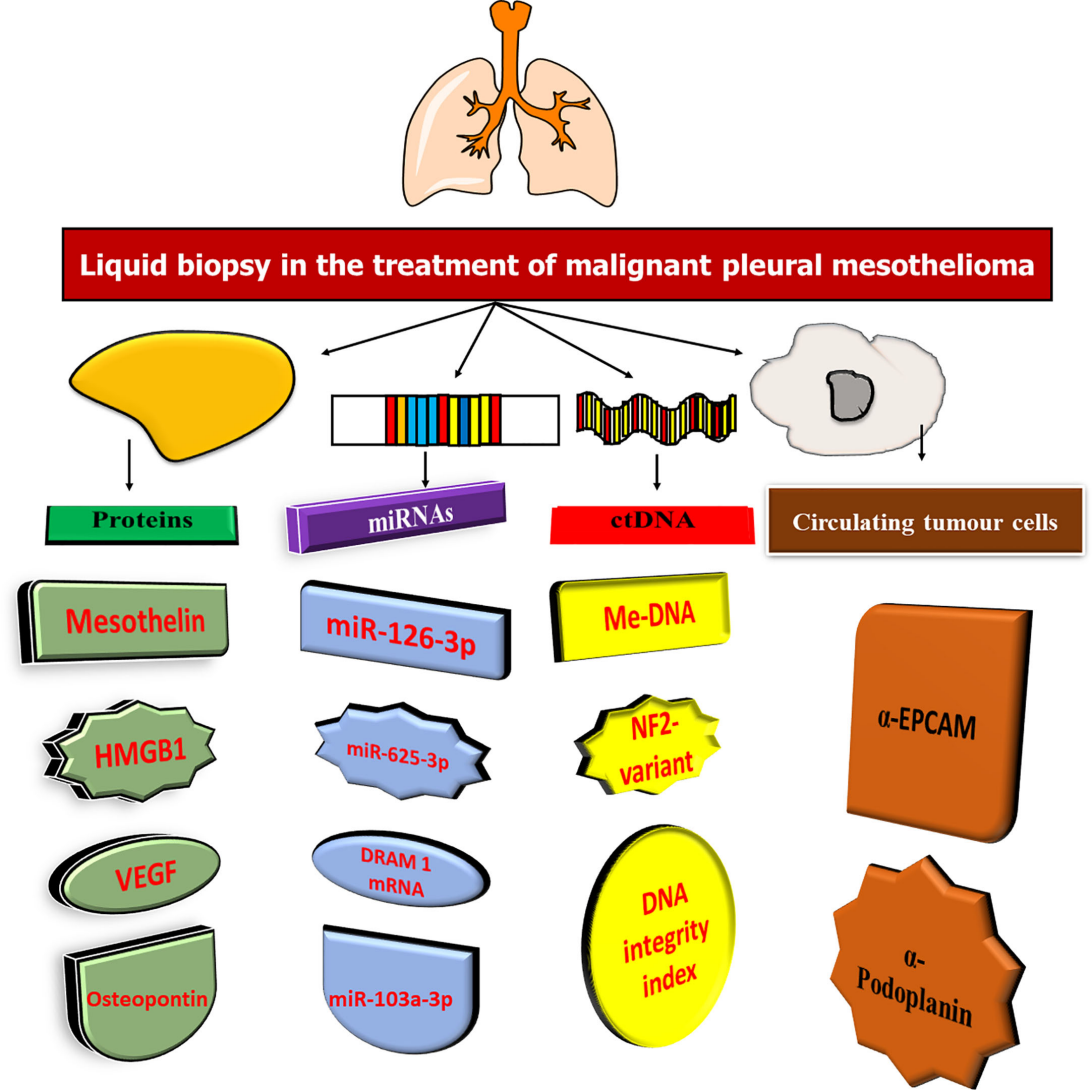
Analysis of systemic circulating biomarkers in the malignant pleural mesothelioma.

In recent decades, cancer biomarkers have been promoted as a more cost-effective method of cancer diagnosis and treatment. In an appropriate clinical context, cancer biomarkers can play an important role in diagnosis, prognosis, and prediction and monitoring of therapeutic response (103). Traditional (glycol)-protein biomarkers have been studied in mesothelioma over the years, primarily as diagnostic or screening biomarkers in case-control settings. Many novel biomarkers for mesothelioma, such as mRNA, miRNA, DNA, and antibody targets, have recently been proposed through high-throughput biomarker discovery initiatives (104). In this situation, blood and pleural effusion are suitable sources of non-invasive biomarkers (105). In addition, cancer cells can release proteins and nucleic acids, which are epigenetically regulated, into circulation, and CTCs and EVs can also be identified (**Figure 4**).

#### 8.8.5 Protein about mesothelin/soluble mesothelin

In ovarian and pancreatic cancers, as well as in sarcomas and multiple myelomas, the cell surface glycoprotein mesothelin is abundant (MM). Variants 1, 2, and 3 are the three isoforms of the virus that can reach the bloodstream. While variation 1 is the most prevalent, the name soluble mesothelin-related protein (SMRP) encompasses all three variants (106). SMRP was shown to be 83% sensitive and 95% specific in patients with multiple myeloma (MM) as opposed to healthy persons, other malignant malignancies, and inflammation of the lung or pleura (107). This may be due to the fact that the study by Robinson et al. added a substantial number of MM and a smaller percentage of numerous other mesothelin-expressing tumors, such as ovarian or pancreatic cancer. SMRP has been associated by others with similar findings. SMRP levels were elevated in only 50% of mesothelioma patients, but were not elevated in sarcomatous cases (108). Thus, a single diagnostic marker could not be justified. This assay uses a sandwich ELISA technique to quantify mesothelin in human blood and pleural fluid and identifies mesothelin variants 1 and 3. Epithelioid and biphasic MPM can be monitored using this test. According to four investigations, individuals with biphasic MPM and advanced-stage epithelioid had considerably greater SMRP quantities than those with early disease, which might represent tumor burden (109). The measuring N-ERC/mesothelin quantities can be determined using the ELISA technique an alternative to the Mesomark™ kit. In addition, N-ERC/mesothelin is claimed to have diagnostic usefulness for MM, with sensitivities of 71-90% and specificities of 88-93%. For monitoring chemotherapy response in MM patients, N-ERC/mesothelin has also been recommended (110). Before chemotherapy, increased SMRP amounts may be used to assess response and progression, as well as to monitor surgical progress. Few articles have demonstrated that SMRP is an independent negative predictor of the patients’ overall survival (OS). In contrast, no predictive value has been demonstrated for the SMRP gene, but Roe et al. found that increased mesothelin expression in tumors was asbestos exposure screening using mesothelin has also been studied, with mixed results. When comparing people with MPM to someone with pleural metastasis of carcinomas or benign pleural lesions, mesothelin levels in pleural fluid have also been considered to be significantly diagnostic. Furthermore, mesothelin levels in the pleural fluid were greater than those in the serum. The level of mesothelin in pleural fluid was considerably greater in epithelioid MPM than in sarcomatous MPM, consistent with the results of SMRP. Contrary to popular belief, measurement of mesothelin in pleural fluid was more sensitive (71% versus 35%) and specific (89% versus 100%) than cytologic examination (111).

#### 8.8.6 Osteopontin

Osteopontin (OPN) is indeed a glycoprotein that aids in the facilitation of cell-to-cell communications and is overexpressed in many malignancies, especially lung, breast, and colon cancers (112). A meta-analysis of serum and plasma OPN published in 2014 found pooled specificity and sensitivity of 0.81 (95 percent confidence interval: 0.79-0.84) and 0.57 (95 percent confidence interval: 0.52-0.61), respectively, with significant heterogeneity across the nine studies. In serum, the presence of thrombin cleavage sites reduces the repeatability of serum measurements. In addition, it appears that serial monitoring of OPN has minimal value. According to the study by Hollevoet et al, osteopontin levels did not decrease after surgery as in patients with SM or MPF. Nevertheless, osteopontin has been shown to play a potential role as a baseline predictor of poor prognosis (100). There are several studies suggesting that high osteopontin levels at disease onset are associated with poor prognosis, regardless of histology. Given the variation in values depending on the ELISA used, future research should use a method based on consensus (113).

#### 8.8.7 Fibulin-3

As a member of the fibulin family (extracellular glycoproteins), Fibulin-3 has been linked to cell proliferation and migration through the EGF-containing fibulin-like extracellular matrix protein-1 (EFEMP-1) gene (114). From 32,000 probe IDs evaluated with the HG1 Affymetrix array, 48 MPMs with matching normal peritoneum showed a 7-fold increase (P=10^-9^) in EFEMP1 RNA expression (100). Individuals having MPM were distinguished from normal asbestos-exposed controls and also patients with other cancers using the plasma Fibulin-3 level. PE Fibulin-3 level could also be detected to distinguish individuals with MPM from individuals with non-MPM pleural effusion (PE), and PE fibulin-3 levels were predictive of MPM. A plasma fibulin-3 threshold of 52.8 ng/mL had a sensitivity of 96.7% and a specificity of 95.5% in patients with MPM. However, a blinded validation cohort from the Princess Margaret Cancer Centre showed significantly lower accuracy. Increased migration, colony formation, and proliferation were seen after EFEMP1 was transfected to ensure effective and efficient mesothelial cells, while the reverse functional features were observed when siRNA FBLN3 was transfected into two MPM cells. However, additional research from Australia and Europe demonstrated Fibulin-3 failed to differentiate individuals with MPM from other diseases. The diagnostic performance of Fibulin-3 was poor compared to mesothelin, despite studies that provided significant support for its use (115).

#### 8.8.8 HMGB1

HMGB1 is a protein molecule and may be found in the nucleus of cells, where it is physiologically expressed with its affinity for TLRs and RAGE. It is involved in the immune response against pathogenic pathogens as well as tissue damage, causing inflammation and cell growth as a result of the binding of Toll-like receptors (TLRs) (116). In addition, modulators impart a crucial role in the development of the MPM. After asbestos exposure, mesothelial cells die, releasing HMGB1 and TNF-α into the extracellular space. Mesothelial cells undergo metamorphosis when the NF-κB pathway is activated. For example, researchers found that MPM patients (n = 61) had considerably greater blood levels of HMGB1 compared to normal people who were exposed to asbestos (n = 45). Despite having a low sensitivity (34.4%), the MPM clinical diagnosis showed the highest specificity and favorable predictive values (PPV) at a cut-off value of 9.0 ng/mL (117). This study further discovered that baseline stage and serum HMGB1 levels both were distinct prognosis factors, implying that HMGB1 might be employed as both a prognostic and diagnostic marker. Napolitano and colleagues showed that hyperacetylated HMGB1 might serve as a diagnostic tool. The researchers found that at a 2.0 ng/mL threshold, they were able to distinguish patients with MPM from those who had been exposed to asbestos and healthy controls with 100% sensitivity and specificity. A hyperacetylated version of HMGB1 was identified in MPM supernatants, which was measured *in-vitro* using mass spectrometry (MS). HMGB1 is passively released from the nucleus of necrotic cells following asbestos-induced necrosis, while the hyperacetylated isoform is actively secreted from the cytoplasm of transformed cells (118).

#### 8.8.9 Circulating tumor DNA

Healthy and malignant cells undergoing apoptosis or necrosis produce circulating free DNA (cfDNA). In contrast, cfDNA is made up mostly of ctDNA that comes from tumor cells and contains somatic mutations; as a biomarker in cancer, it has the potential to be used for a wide range of purposes, such as staging and prognosis, tracking therapy response or minimal residual disease (MRD), and identifying the associated acquired resistance pathways (119). The dubbed integrity index, a biomarker predicated on the proportion of long to short cfDNA fragments, has been established. The pleural fluid DNA integrity index was greater in MPM (n = 52) than that in benign effusions (n = 23) (120). A high pleural fluid DNA integrity index was shown to have a positive predictive value (PPV) of 81 percent for the detection of MPM in patients with cytologically negative pleural effusion, suggesting that it can be used to guide more invasive treatments. The discovery of ctDNA in the management of MPM patients has opened new possibilities. Additionally, used whole- exome sequencing (WES) to detect cancer-specific variations in germline and tumor DNA from 10 individuals with MPM. Droplet digital PCR at allelic fractions ranging from 0.28 to 0.9 percent detected three of five treatment-naive individuals (a detection rate of 60%). Because ctDNA might be employed as a biomarker for treatment response assessment (121). In order for ctDNA analysis to be widely used in daily clinical practice, it is necessary to validate these results and utilize a technology that is accessible.

#### 8.8.10 Heparanase

The intercellular matrix (ICM) is important for regulating tissue stability and function. In the ICM, heparan sulfate proteoglycans orchestrate critical connections and transmit signals that control cellular expansion and maturation (122). The main enzyme for intercellular heparan sulfate degradation is heparanase, and malignant tumors exhibit a dramatically increased expression of this enzyme (123). Heparanase impact on tumorigenesis was observed in pre-clinical *in-vivo* murine prototypes of MPM in sensitivity to heparanase-inhibiting drugs and heparanase gene ablation. Diagnostically, individuals with greater concentrations of immunoreactivity for heparanase had shorter survival times than those with lower concentrations of the enzyme. The diagnostic and therapeutic outcomes are validated by the heparanase blocker’s notable potential to limit the development of orthotopically implanted mesothelioma tumor xenografts. Significantly, the heparanase blockers *i.e* defibrotide and pixatimod (PG545), which are frequently used as mesothelioma chemotherapeutics, seemed more efficient than cisplatin at suppressing tumor development. These findings were strongly related to a radically longer life of mesothelioma-mediate mice (124).

#### 8.8.11 STAT3

A transcriptional variable called STAT3 (Signal transducer and activator of transcription 3) regulates genes associated with controlling longevity, and multiplication when specific receptors for growth factors or cytokines are stimulated (125). Downregulation STAT3 stimulation is a key mechanism that promotes tumorigenesis in blood cancer and multiple solid tumors, frequently through stimulating tyrosine kinases (126). A higher incidence of STAT3 stimulation was observed in MPM, and tyrosine phosphorylation of the STAT3 gene was detected in 61.4% of the recorded cases (127). Approaching transcription of the STAT3 gene could have beneficial effects by increasing functional immunological activity and reducing immunological evasion responses. Upregulation of inducible T-cell costimulatory ligand (ICOSLG) and IL-8 was seen when the STAT3 cascade was targeted. Atovaquone and pyrimethamine, STAT3 cascade antagonists, provide compelling evidence that STAT3 plays an immunosuppressive and growth-enhancing activity in MPM, establishing STAT3 as a prospective pharmacological candidate (128).

### 8.9 Adoptive T-cell treatment

Adoptive T-cell treatment, a remarkable pharmacological approach, significantly increases the number of T cells that may interact with tumor antigens. Optimizing the functioning of regulatory immune cells is a potential strategy to support tumor eradication since, typically, during tumor progression, T-cell tracking of autoantigens may be impaired and may obstruct the subsequent death of tailored cells (129). Several approaches to this treatment have been developed over the years, but the production of T cells with chimeric antigen receptors (CAR -T) has been shown to be most effective. Mesothelin indicates the strongest promising antigen candidate in mesothelioma malignancies due to its upregulation and apparent correlation with tumor activity (130). Mesothelin CARs are reportedly being investigated in a number of phases I clinical testing, which are sponsored by robust exploratory preclinical assessments. The mRNA electroporation approach was used to establish T cells with CAR transcription and it resulted in a potent antitumoral action in xenograft designs of living MPM. Following these encouraging results, a phase I clinical trial (NCT01355965) was initiated to investigate the efficacy and viability of modified T cells encoding mesothelin CAR, (131). Another experiment (NCT02159716) was also conducted to examine a lentivirus reprogramming vector encoding anti-mesothelin 2^nd^-generation CAR; although adoptive T-cells were accepted satisfactorily in this test, the findings were not greatly advanced (130). A new experiment (NCT03054298) is attempting to circumvent the challenge of dissemination by intratumorally injecting exclusively human anti-mesothelin CAR -T (132).

### 8.10 Cancer vaccines

Cancer vaccines focus on exploiting the distinct features of antigen-presenting cells to augment Th-cell activation and, as a result, stimulate cytotoxic regulatory T-cells. Dendritic cells (DCs) are extensively being explored as vaccine additives because of their special potential to trigger CD8 T-cell invasion, and have been documented to be associated with improved total longevity in mesothelioma individuals (133). Developed DCs are challenged by an endogenous tumor cell homogenate and cytokine combination, a significant origin of antigens (referred to as the MesoPher vaccine), and as a result, they may trigger phenomenal regulatory T-cell responses targeting cancerous clones, as evidenced in a murine paradigm of mesothelioma. Positive preclinical findings prompted investigators to conduct a preliminary clinical study with nine subjects, five of whom received pulsing DCs following chemotherapeutics, while the other four were untreated. Preliminary outcomes revealed the therapeutics tolerability and efficacy, and no significant toxicities were reported (134). Systematic observations cannot be established from this investigation because of the small number of volunteers, but it enabled facilitated the development of a controlled phase 2/3 study (NCT03610360) to investigate the effectiveness of DCs encapsulated with allogenic tumor homogenate in MPM individuals undergoing first-line treatment. To possibly explore DCs as a novel therapeutic choice, comprehensive fundamental conclusions will be monitored (135). Wilms tumor protein (WT1) has been investigated as a cancer vaccine candidate in the domain of mesothelioma therapy because of its potential to activate CD8 WT1 and CD4-targeted responses. A controlled phase 2 investigation (NCT01265433) has been designed to evaluate the augmented effectiveness of WT1 analog vaccination following a multifunctional treatment; intriguingly, it was conducted in a prototype study with 9 subjects to initially examine its effectiveness and tolerability. Cancer vaccinations are an interesting approach because experimental outcomes show that they are efficacious as well as safe, but more exploration and multicenter clinical studies are anticipated before they can be approved for use in the frontline context (136).

### 8.11 Emerging mesothelioma-based specific therapy

The new mesothelioma targeting has been used to provide promising alternatives for therapeutic application, although several immunotherapies are currently being explored. Some of these are presented in the following sections and reinforce the idea that understanding tumor physiology is essential for the logical development of unique, tailored treatment approaches.

#### 8.11.1 VISTA inhibitor

Immunologic proteins are the target of much attention because of their role in the immunosuppressive tumor milieu that is characteristic of solid malignancies like mesothelioma. They can impair immune response activity by interacting selectively with immune cells, which prevents the immune system from performing its defensive activity. V-domain immunoglobulin (Ig) suppressor of T cell activation (VISTA) is one of the newly revealed proteins that function by inhibiting T cells’ cytokines release and their multiplication. Mesothelium has been shown to have increased expression of VISTA and a phase I clinical trial (NCT02812875) is currently underway to evaluate the efficacy of an antagonist against VISTA in individuals with advanced lymphomas or solid tumors (137, 138).

#### 8.11.2 TIM-3 inhibitor

Immune system cells like DC, macrophages, CD4 and CD8 T cells are the major sources of T-cell immunoglobulin mucin 3 (TIM-3), which triggers inhibition of the Th1 activity and increases the recruitment of regulatory T-cells (139). The research has proven that TIM-3 is frequently expressed in programmed death ligand-1 mesothelioma tumors and that better longevity after the anti-CTLA4 therapies is attributed to reduced transcription of TIM-3; as an outcome, determining the involvement of TIM-3 may be a plausible prognostic variable to anticipate patients who will respond to therapy (140). Clinical trials are currently exploring many therapeutics targeting this distinctive biomarker for the treatment of metastatic solid tumors, either alone or in combination with conventional cancer immunotherapies; beyond that, results are not yet available and the potential benefits are still unclear (138).

#### 8.11.3 LAG-3 inhibitor

Lymphocyte activation gene-3 (LAG-3) is an inhibitory receptor that can restrict T cell activity and proliferation, therefore maintaining immunological regulation. LAG-3 is not explicitly produced by cancer cells, whereas it is frequently observed in the pleural edema of mesothelioma individuals and tumor-invading lymphocytes (141). Inhibition of LAG-3 is now being evaluated in clinical testing for the management of various cancers, such as breast carcinoma. The data are promising, with survival rates of 50%, which would be a compelling rationale for using this approach in mesothelioma therapy (138).

#### 8.11.4 TLR-9 agonist

Toll-like receptor 9 (TLR-9) is a suitable potential option for achieving substantial immunological stimulation at the tumor location. It is a cytoplasmic DNA receptor that is stimulated by DNA binding and can therefore initiate a mechanism that enhances signaling pathway expression such as AP-1 and NF-κB. It can augment innate immunity by circulating cytokines and maturing DCs. As a result, TLR-9 agonists may represent ideal alternatives for triggering an immune system response that will fight cancer (142).

## 9 Recent clinical evidence of mesothelioma

Malignant mesothelioma is a rare and fatal cancer that mainly affects the pleura and peritoneum (143). The prevalence of mesothelioma is expected to increase worldwide and existing therapies are inadequate. **Table 1** shows various clinical trials for the treatment of mesothelioma. Detailed discussion of recent clinical evidence is discussed below:

**Table 1:** Various clinical trials involved in the treatment of mesothelioma (Data was retrieved from ClinicalTrials.gov site with search terms like treatment, vaccines, and mesothelioma on May 15, 2023).

Title	Trial Phase	Status	NCT number	Intervention	Trial Year and number of participants (n)	Results	Locations	Adverse effects
Chemotherapy with Eloxatin^®^ and gemcitabine for mesothelioma	Phase 2	Completed	NCT00859469	Oxaliplatin (platinum-based chemotherapeutic) and gemcitabine (antimetabolite)	April 2004; n= 29	In patients with MPM, an open-label, phase 2 investigation finished in 2013 assessed the effectiveness of gemcitabine and oxaliplatin combined therapy. The trial is anticipated to enlist a total of 29 individuals, and each of them will enroll for 6 months. The results revealed that the prognosis of 24 individuals was graded as poor. The safety profiles of the drugs were satisfactory, while the remaining individuals had a persistent illness or had only partially responded	U.S. Columbia University Medical Center New York	Alopecia, thrombocytopenia, leukopenia, peripheral neuropathy, and dyspnea
Mesothelioma Treatment Using Tomotherapy	Phase 2	Completed	NCT00469196	Tomotherapy	October 2006; n= 18	Results not mentioned	Cross Cancer Institute Edmonton, Alberta, Canada	Nausea, vomiting, alopecia, skin irritation, and fatigue
Metastatic Mesothelioma: A Single-Dose FMT Infusion as an Adjuvant to Keytruda	Early Phase 1	Completed	NCT04056026	Fecal microbiota transplant (FMT)	18^th^ September 2018; n= 01	Results not mentioned	ProgenaBiome Ventura, California, United States	Abdominal pain, constipation, bloating, transient diarrhea, and bacteremia.
Metastatic Mesothelial Cancer and Serum Biomarkers	Not Applicable	Completed	NCT02029105	Hyaluronan, syndecan-1, mesothelin, and osteopontin	January 2004; n= 230	Results not mentioned	Research and Application Center for Lung and Pleural Cancer in Eskisehir, Turkey	Nausea, neutropenia, keratopathy, peripheral neuropathy, and atrial fibrillation
Mesothelioma Patients with 11C-Methionine PET/CT Imaging	Not Applicable	Completed	NCT02519049	–––	September 2004; n= 30	Results not mentioned	Istituto Clinico Humanitas Rozzano, Milano, Italy	Claustrophobia, pain, and allergic reactions
Preliminary evaluation of malignant peritoneal mesothelioma prognostic biomarkers using prospectively collected pleural	Not Applicable	Recruiting	NCT03683680	Claudin-15 (CLDN15)/Vimentin (VIM) and mesothelioma prognostic test	31^st^ October 2018; n= 240	Results not mentioned	The Women’s Hospital of Boston, The city of Boston, Massachusetts, USA	—
Anti-PD-1 Antibody Pembrolizumab in Patients With Resectable Malignant Pleural Mesothelioma: A Pilot Study	Phase 1	Recruiting	NCT02707666	Pemetrexed (antimetabolite), cisplatin (platinum-based chemotherapeutic), and pembrolizumab (PD1 checkpoint inhibitor)	25^th^ February 2016; n= 15	Results not mentioned	The University of Chicago, Illinois, United States	Abdominal pain, joint pain, loss of appetite, allergic reaction, and dyspnea
Mesothelioma Vaccine Poly-ICLC (Hiltonol^®^)	Phase 1	Recruiting	NCT04525859	–––	19^th^ August 2020; n= 19	Results not mentioned	Mount Sinai’s Icahn School of Medicine, New York City	Muscle pain, cough, fatigue, and dyspnea
HRD Malignant Mesothelioma Patients Utilizing Olaparib	Phase 2	Recruiting	NCT04515836	Olaparib (PARP inhibitor)	19^th^ February 2021; n= 56	Results not mentioned	Medical Center at the University of Chicago Cities in the United States, Chicago	Asthenia, headache, loss of appetite, constipation, and abdominal pain
Combination of Pembrolizumab and Lenvatinib in second- and third-line malignant pleural mesothelioma	Phase 2	Recruiting	NCT04287829	Lenvatinib (Kinase inhibitor) and pembrolizumab (PD1 checkpoint inhibitor)	1^st^ March 2021; n= 58	Results not mentioned	Antoni van Leeuwenhoekziekenhuis (NKI-AVL) Amsterdam, Noord-Holland, Netherlands	Stomatitis, weight loss. hair loss, loss of appetite, and joint pain
Treatment of Malignant Pleural Mesothelioma with Accelerated Hypofractionated Radiotherapy	Not Applicable	Recruiting	NCT03269227	Accelerated hypofractionation with Tomography	14^th^ August 2017; n= 30	Results not mentioned	Meldola, Italy’s SC Radiation Therapy Centers	Chest pain, esophagitis, cough, and dyspnea
Evaluating Surgery After Radiation Therapy to Remove Pleural Tissue in Mesothelial Cancer Patients	Not Applicable	Active, not recruiting	NCT04028570	Radiation therapy	5^th^ September 2019; n= 12	Results not mentioned	Princess Margaret Hospital, University Health Network Toronto, Ontario, Canada	Fatigue, alopecia, headache, and lymphedema
Patients with malignant pleural mesothelioma receive Pembrolizumab with hypofractionated stereotactic	Phase 1	Recruiting	NCT04166734	Stereotactic body radiation and pembrolizumab (PD1 checkpoint inhibitor)	26^th^ January, 2021; n= 18	Results not mentioned	NHS Foundation Trust Chelsea and Beatson West of Scotland Cancer Centre Glasgow, United Kingdom	Loss of appetite, musculoskeletal pain, hyponatremia, and vitiligo
MesomiR 1: A Phase 1 study of TargomiRs as 2^nd^ or 3^rd^ line treatment for patients with recurrent MSM and non-small cell lung cancer	Phase 1	Completed	NCT02369198	miR-16 Mimic (TargomiRs)	September 2014; n= 27	Results not mentioned	Northern Cancer Institute Sydney, New South Wales, Australia	—
Mesothelioma Stratified Therapy (MiST): A Multi-drug Phase 2 trial in malignant mesothelioma	Phase 2	Active, not recruiting	NCT03654833	Abemaciclib (CDK4/6 inhibitor)	28^th^ January 2019; n= 186	Results not mentioned	University Hospitals of Leicester NHS Trust, United Kingdom	—
Patients with mesothelin-positive pleural mesothelioma receive Pembrolizumab with or without anetumab ravtansine	Phase 1/2	Active, not recruiting	NCT03126630	Anetumab ravtansine (mesothelin-targeted antibody-drug conjugate) and pembrolizumab (PD1 checkpoint inhibitor)	8^th^ February 2018; n= 110	Results not mentioned	Mayo Clinic in Arizona Scottsdale, Arizona, United States	Peripheral sensory neuropathy, keratitis, corneal microdeposits, and neutropenia

144 examined the role of calcitriol for its possible anticancer function in malignant pleural mesotheliomas (MPM). Both cytoplasmic and nuclear VDR is expressed in human MPM cell lines, and calcitriol reduced cell survival and proliferation; in addition, calcitriol enhanced the inhibitory effect of the chemotherapeutic agent. c-Myc and other cell cycle regulators were inhibited, and cell cycle progression was arrested. An altered mitochondrial activity and suppression in the development of the respiratory chain complex subunits were seen when MPM cells were exposed to calcium calcitriol. Calcitriol also inhibited the viability of human MPM cells. It’s possible that vitamin D derivatives, alone or in conjunction with chemotherapy, might be used to treat MPM (144).

145 investigated BAP1 inactivating mutations to promote PARPI sensitivity in the mesothelioma, and blend treatment with temozolomide (TMZ) might be helpful. On the basis of BAP1 mutation status, nuclear localization, protein expression, as well as the PARPIs, talazoparib and olaparib alone or in combination with TMZ in the cell lines obtained from the 10 patients. Using ubiquitin with 7-amido-4-methylcoumarin, the deubiquitinase activity of BAP1 was determined. CRISPR-Cas9 was used to create mesothelioma cell lines that lack BAP1. O6-methylguanine-DNA methyltransferase and Schlafen 11 (SLFN11) also promote response to PARPIs and TMZ, therefore we examined their expression and association with drug response. The results revealed that all 10 cell lines have BAP1 mutations or copy-number changes, or both. The DUB activity was intact in four cell lines, while BAP1 was localised in two. Both olaparib and talazoparib were classified as sensitive (two) or resistant (seven) cells based on their half-maximum inhibitory doses, which varied from 4.8 mM to higher than 50 mM and 0.039 mM to greater than 5 mM respectively. However, talazoparib sensitivity was not increased in cell lines with BAP1 knockouts. Cells with little or no MGMT expression were more sensitive to PARPI when combined with temozolomide (145).

146 performed a functional study of microRNAs and HEG1 utilising MM cell lines (H2052, MESO4 and H226). After temporary transfection with microRNA-23b (miR-23b) inhibitor and/or HEG1 siRNA, the MTS test indicated a substantial reduction in cell growth. Annexin V assay demonstrated that inhibition of miR-23b and/or HEG1 resulted in apoptosis. It was shown that suppression of miR-23b and/or HEG1 led to an increase in autophagy-related protein LC3- II.According to these findings, miR-23b contributes to HEG1-dependent cell proliferation in MM cells by evading cytotoxicity produced by apoptosis and autophagy. Because HEG1-dependent and/or miR-23b facilitated miR-23b signalling may be an important target for the diagnosis and treatment of the MM (146).

Johnson et al., 2020 examined the alteration in the gene expression leading by YB-1 knockdown in the three mesothelioma cell lines, they utilized an unbiased RNA-seq method (REN, VMC23 and MSTO-211H cells). It was shown 150 common genes including those involved in mitosis, extracellular matrix organisation and integrin regulation were found to be impacted by YB-1 knockdown. However, apart from these differences, each of the cell lines also showed different gene expression profiles that were significantly enriched. An interesting difference between the cell lines was the dysregulation of the STAT3 and P53 pathways. Using apoptosis assays and single-cell time-lapse imaging, we found that the REN, VMC23, and MSTO-211H cells exhibited either enhanced G1 arrest, cell death, or abnormal mitotic division. These results indicate that knockdown of YB-1 affects a key set of genes in mesothelioma cells. Depending on the STAT3/p53-pathways and the genetic structure of the cell, YB-1 deletion reduces mesothelioma growth (147).

148 performed proteome studies of the liver to uncover molecular drivers of that organotropism and possible treatment targets (M5-T1). The SWATH-MS working prototype trends of the liver from healthy rats (G1), nearby non-tumorous liver of controlled tumour-bearing rats (G2), and curcumin-treated liver of rats (G3) without hepatic metastases were found to have quantitative differences. Consequently, in G3, 12 biomarkers were indicating enhanced immunological response to M5-T1 cells, and 179 liver biomarker abnormalities (G2 vs. G3) (G3 vs. G1). There are seven unique biomarkers for the M5-T1 tumor when these 179 possibilities are compared with proteins that exhibit changes in abundance associated with increased invasiveness in four different rat mesothelioma tumor models. Further connections in between 7 biomarkers, the key immunological biomarker being purine nucleoside phosphorylase, and other proteins have shown a complicated network that governs liver colonization and treatment efficiency. Results from this study suggest that sarcoma cells and highly invasive cancer cells with sarcomatoid phenotypes are related (148).

The DENIM experiment (NCT03610360) is evaluating dendritic cells (DC) encapsulated with allogenic tumor cell lysate in a controlled phase 2-3 investigation. The DC is administered to patients as a continuous treatment in addition to the best supportive care following chemotherapy, or BSC alone, depending on the outcome of a randomization procedure. Overall survival (OS) is the main outcome of this investigation. The cellular membrane biomarker mesothelin, which is primarily expressed in mesothelioma, has also been the subject of investigations. These incorporate CAR-T cells and an anti-mesothelin antibody with or without a pharmacological combination. The final has been investigated in a phase 1 experiment in conjunction with pembrolizumab, attaining a disease control rate (DCR) of approximately 60% (149, 150).

In light of an earlier phase 2 investigation that reported a DCR of 87.5% and a response rate of 25%, the INFINITE research (NCT03710876), which is in its phase 3, is examining the performance and safety of intrapleural delivery of adenovirus-administered interferon α-2b when combined with gemcitabine and celecoxib (orally). Gemcitabine and celecoxib were administered orally to the control group only. The patients must have undergone a minimum of one comprehensive treatment before. OS is the main outcome of this investigation. Among patients lacking the option of inserting an intrapleural tube, this strategy might not be practical (151).

Opportunities for personalized therapy in mesothelioma have been discovered through superior knowledge of genomic modification, alterations, and predominant biology (152). A genomic stratification-based approach to therapy is being evaluated in the MiST study. The therapy with a combination of a poly ADP ribose polymerase (PARP) blocker with Ig antibody for the platin-sensitive condition, a vascular endothelial growth factor (VEGF) blocker with a programmed cell death ligand-1 (PDL1) blocker for PDL1 upregulation positive conditions, an AXL blocker combined with a PD1 blocker with no unique biomarker, a cyclic dependent kinase (CDK4/6) blocker for p16ink4A-negative conditions, and PARP blockers for BAP1/BRCA1 negative conditions are all included in the multiple arm assignment (152). In patients with BAP1 or BRCA1 deficits, the initial arm of the research (MiST1) with rucaparib reportedly achieved its principal goal (DCR of 23% and 58% at 24 weeks and 12 weeks, respectively). Rucaparib was highly accepted, with only 9% of patients experiencing level 3/4 toxicity. Additional research with PARP blockers is necessary for mesothelioma with translational recombination deficit linked to a deletion in BRCA1 (14). Abemaciclib was administered to treat 26 patients with p16ink4A-deficient mesothelioma in the newly presented MiST2 investigation (153). The main goal was achieved since DCR at 12 weeks was reported by 14 patients (56%). 12% of patients had grade 4 or greater adverse events (AE), 23% reported severe AE, and 1 patient succumbed from febrile neutropenia. This work will contribute significant insights to the development of tailored mesothelioma therapy due to genomic stratification (153).

Arginine depletion has been proven to be a potential treatment for individuals with impairment of argininosuccinate synthetase 1 (ASS1) and may be accomplished with pegylated arginine deiminase (ADI-PEG20). In the non-epithelioid category, ASS1 depletion is typical. A phase 3 experiment is currently evaluating chemotherapy with or without ADI-PEG20 (NCT02709512) in 386 patients following promising clinical outcomes in a phase 2 experiment. Tazemetostat (Zeste- Homolog 2 methyltransferase inhibitor) is a new approach to selective treatment for BAP1-mutated mesothelioma (154, 155).

Solid malignancies, such as ovarian, pancreatic, and mesothelioma tumors, express mesothelin, a transmembrane tumor antigenic substance, strongly. In mesothelioma, it has been explored as a potential pharmacological target, especially antibody-drug conjugate (ADC) and CAR-T therapy. In a phase 1 investigation, an anti-mesothelin-specific ADC called anetumab ravtansine was found to have promising antitumor efficacy and a tolerable safety record in mesothelioma patients who had already received treatment. Pembrolizumab is now being investigated with or without anetumab ravtansine (NCT03126630) in phase 1/2 research for mesothelin-positive pleural mesothelioma (156).

## 10 Conclusion

The mesothelium plays an important role in the development of organs in the embryonic coelom, such as the heart, lungs, and intestine. Various diseases can damage mesothelium-derived structures, with mesotheliomas being the most serious. Various genes characteristic of growing and existing mesothelial structures are also very useful as markers in the diagnosis of mesothelioma and allow differentiation from malignant mesothelioma. There are still new cases of MPM being diagnosed even though the use of asbestos has been banned in many countries. This is because asbestos exposure is the most common cause of MPM. However, there are other factors that mean this disease has lagged behind other cancers in the development of new treatments, leaving patients with few treatment options. There remains an urgent need for novel and effective treatment modalities, although drugs targeting immune checkpoints and angiogenesis have shown promising effects.

## Author contributions

Conceptualization, BB and RS. Data collection, SR, AJ and SN. Formal analysis, HA-G and AS. Writing—original draft preparation, BB, SN, RW, RJ, RS and WE. Writing—review and editing, JK, RS, MG and SB. Supervision, RS. All authors contributed to the article and approved the submitted version.
